# Supplementary material for: Cost-effectiveness of the ReDIRECT/counterweight-plus weight management programme to alleviate symptoms of long COVID
Source: Nat Commun. 2025 Jul 1;16:5592. doi: 10.1038/s41467-025-59909-6 (PMC12218384; doi:10.1038/s41467-025-59909-6)
Supplement: Supplementary file 1 — Supplementary Information [file 41467_2025_59909_MOESM1_ESM.pdf]

Supplementary Materials: Cost-Effectiveness of the ReDIRECT/Counterweight-Plus Weight Management Programme to Alleviate Symptoms of Long COVID

Contents

CHEERS 2022 Checklist 2
Data collection instruments 5
Work and Productivity Impairment Questionnaire 5
Personal Food and Drink costs questionnaire 7
Intervention Micro-Costing 8
Outlier Sensitivity Analysis 8
References 12

CHEERS 2022 Checklist

Table S1. CHEERS 2022 Checklist [1]

Topic	No.	Item	Location where item is reported
**Title**			
	1	Identify the study as an economic evaluation and specify the interventions being compared.	Title page
**Abstract**			
	2	Provide a structured summary that highlights context, key methods, results, and alternative analyses.	Abstract
**Introduction**			
**Background and objectives**	3	Give the context for the study, the study question, and its practical relevance for decision making in policy or practice.	Introduction section
**Methods**			
**Health economic analysis plan**	4	Indicate whether a health economic analysis plan was developed and where available.	Methods section, 'Economic Evaluation' sub-heading
**Study population**	5	Describe characteristics of the study population (such as age range, demographics, socioeconomic, or clinical characteristics).	Methods section, 'The main randomised controlled trial (ReDIRECT)' subheading, last paragraph and Results section 'baseline characteristics' subheading, Table 1.
**Setting and location**	6	Provide relevant contextual information that may influence findings.	Methods section, 'The main randomised controlled trial (ReDIRECT)' subheading, last paragraph
**Comparators**	7	Describe the interventions or strategies being compared and why chosen.	Methods section, 'The main randomised controlled trial (ReDIRECT)' subheading, second paragraph
**Perspective**	8	State the perspective(s) adopted by the study and why chosen.	Methods section, 'Economic evaluation' subheading
**Time horizon**	9	State the time horizon for the study and why appropriate.	Methods section, 'Economic evaluation' subheading
**Discount rate**	10	Report the discount rate(s) and reason chosen.	Not applicable - time horizon is less than 1 year

Topic	No.	Item	Location where item is reported
**Selection of outcomes**	11	Describe what outcomes were used as the measure(s) of benefit(s) and harm(s).	Methods section, 'Health outcome for the economic evaluation' subheading
**Measurement of outcomes**	12	Describe how outcomes used to capture benefit(s) and harm(s) were measured.	Methods section, 'Health outcome for the economic evaluation' subheading
**Valuation of outcomes**	13	Describe the population and methods used to measure and value outcomes.	Methods section, 'Health outcome for the economic evaluation' subheading
**Measurement and valuation of resources and costs**	14	Describe how costs were valued.	Methods section, 'Resource Use' subheading, 'intervention costs', 'healthcare service resource use', 'productivity costs and personal food costs' paragraphs
**Currency, price date, and conversion**	15	Report the dates of the estimated resource quantities and unit costs, plus the currency and year of conversion.	Methods section, 'Resource Use' subheading, first paragraph
**Rationale and description of model**	16	If modelling is used, describe in detail and why used. Report if the model is publicly available and where it can be accessed.	Methods section, 'Missing data' subheading
**Analytics and assumptions**	17	Describe any methods for analysing or statistically transforming data, any extrapolation methods, and approaches for validating any model used.	Methods section, 'Missing data' subheading, 'Incremental cost-effectiveness analysis' subheading
**Characterising heterogeneity**	18	Describe any methods used for estimating how the results of the study vary for subgroups.	Not applicable
**Characterising distributional effects**	19	Describe how impacts are distributed across different individuals or adjustments made to reflect priority populations.	Not applicable
**Characterising uncertainty**	20	Describe methods to characterise any sources of uncertainty in the analysis.	Methods section, 'Incremental cost-utility analysis' subheading
**Approach to engagement with patients and others affected by the study**	21	Describe any approaches to engage patients or service recipients, the general public, communities, or stakeholders (such as clinicians or payers) in the design of the study.	PPIE involvement in the trial design and implementation fully described in the main trial outcomes paper (referenced in our paper)
**Results**			

Topic	No.	Item	Location where item is reported
**Study parameters**	22	Report all analytic inputs (such as values, ranges, references) including uncertainty or distributional assumptions.	Methods section, 'Missing data' subheading
**Summary of main results**	23	Report the mean values for the main categories of costs and outcomes of interest and summarise them in the most appropriate overall measure.	Results section, Incremental cost-effectiveness results (Table 4), Summary cost results (Table 3), summary utility results (Table 2)
**Effect of uncertainty**	24	Describe how uncertainty about analytic judgments, inputs, or projections affect findings. Report the effect of choice of discount rate and time horizon, if applicable.	Figure 5 ICER Plane scatter plots and CEACs, Table 5 (95% CIs).
**Effect of engagement with patients and others affected by the study**	25	Report on any difference patient/service recipient, general public, community, or stakeholder involvement made to the approach or findings of the study	PPIE involvement in the trial design and implementation fully described in the main trial outcomes paper (referenced in our paper)
**Discussion**			
**Study findings, limitations, generalisability, and current knowledge**	26	Report key findings, limitations, ethical or equity considerations not captured, and how these could affect patients, policy, or practice.	Discussion section
**Other relevant information**			
**Source of funding**	27	Describe how the study was funded and any role of the funder in the identification, design, conduct, and reporting of the analysis	‘Acknowledgements’ section, after References
**Conflicts of interest**	28	Report authors conflicts of interest according to journal or International Committee of Medical Journal Editors requirements.	‘Competing Interests Statement’ section, after References

Data collection instruments

Work and Productivity Impairment Questionnaire

**Work Productivity and Activity Impairment Questionnaire:
General Health V2.0 (WPAI:GH)**

The following questions ask about the effect of your health problems on your ability to work and perform regular activities. By health problems we mean any physical or emotional problem or symptom. *Please fill in the blanks or circle a number, as indicated.*

1. Are you currently employed (working for pay)? ____ NO ____ YES
   If NO, check "NO" and skip to question 6.

The next questions are about the **past seven days**, not including today.

2. During the past seven days, how many hours did you miss from work because of your health problems? *Include hours you missed on sick days, times you went in late, left early, etc., because of your health problems. Do not include time you missed to participate in this study.*

   _____HOURS

3. During the past seven days, how many hours did you miss from work because of any other reason, such as vacation, holidays, time off to participate in this study?

   _____HOURS

4. During the past seven days, how many hours did you actually work?

   _____HOURS *(If "0", skip to question 6.)*

5. During the past seven days, how much did your health problems affect your productivity while you were working?

Think about days you were limited in the amount or kind of work you could do, days you accomplished less than you would like, or days you could not do your work as carefully as usual. If health problems affected your work only a little, choose a low number. Choose a high number if health problems affected your work a great deal.

Consider only how much health problems affected productivity while you were working.

Health problems had no effect on my work	0	1	2	3	4	5	6	7	8	9	10	Health problems completely prevented me from working

CIRCLE A NUMBER

6. During the past seven days, how much did your health problems affect your ability to do your regular daily activities, other than work at a job?

By regular activities, we mean the usual activities you do, such as work around the house, shopping, childcare, exercising, studying, etc. Think about times you were limited in the amount or kind of activities you could do and times you accomplished less than you would like. If health problems affected your activities only a little, choose a low number. Choose a high number if health problems affected your activities a great deal.

Consider only how much health problems affected your ability to do your regular daily activities, other than work at a job.

Health problems had no effect on my daily activities	0	1	2	3	4	5	6	7	8	9	10	Health problems completely prevented me from doing my daily activities

CIRCLE A NUMBER

Personal Food and Drink costs questionnaire

**3. Personal food and drink costs**<br><br>**Please think about <u>last week's</u> food and drink purchases for your household and tell us the approximate cost.**	**Weekly cost to you/ your household**<br><br>**If £0 please add '0'**
Food and non-alcoholic drinks (e.g supermarket shopping)	**£**
Alcoholic drinks e.g. wine & beer	**£**
Takeaway meals and snacks eaten **at home** e.g. pizza delivery	**£**
Meals, snacks and drinks **consumed away from home** (e.g. café or restaurant)	**£**
**How many people are there in your household (including yourself) that would be covered by the food and drink costs above?**	**____ adults**<br>**____ children (aged <18)**
**4. All other weekly personal lifestyle costs e.g. exercise classes/gym membership or attendance etc**	**Weekly cost to you £________**

Intervention Micro-Costing

Table S2. Intervention micro-costing

	Mean units per participant (SD)	Unit cost in £ (Source)	Mean cost per participant in £ (SD)
Sachets issued	324 (129)	1.61[a] (Counterweight [2])	522 (208)
Phone calls (measured in minutes)	176 (152)	1.13[b] (PSSRU [3])	200 (173)
Video calls (measured in minutes)	142 (193)	1.13[b] (PSSRU [3])	161 (219)
Chat message (measured in minutes)	1.27 (4.80)	1.13[b] (PSSRU [3])	1.44 (5.44)
All contact (measured in minutes)	320 (184)	1.13[b] (PSSRU [3])	362 (208)
Application development	1 (0)	1[c] (Literature [4])	1.00 (0)
Total cost per participant			885 (340)

a. Assuming £45 per week for 28 sachets (4 sachets per day) = £1.607 per sachet [2]
b. Cost per minute of time for dietitian /coach, assuming cost per working hour = £68 for a Band 7 practice nurse [3]
c. Assuming the application was already developed by Counterweight-Plus for existing interventions [4]
PSSRU: Personal Social Services Research Unit

Outlier Sensitivity Analysis

Due to high baseline resource use attributed to one individual in the control group, it was deemed appropriate to conduct a sensitivity analysis with this outlier removed, to understand the impact (if any) of this outlier resource use on the cost-effectiveness results.

Figure S1 shows healthcare resource use costs by trial arm with the outlier removed, in order to demonstrate between group differences at each time point, in the absence of the outlier. While the mean baseline costs are now more similar at baseline, the between-group difference in mean resource-use cost at 3 months is maintained with the outlier removed.

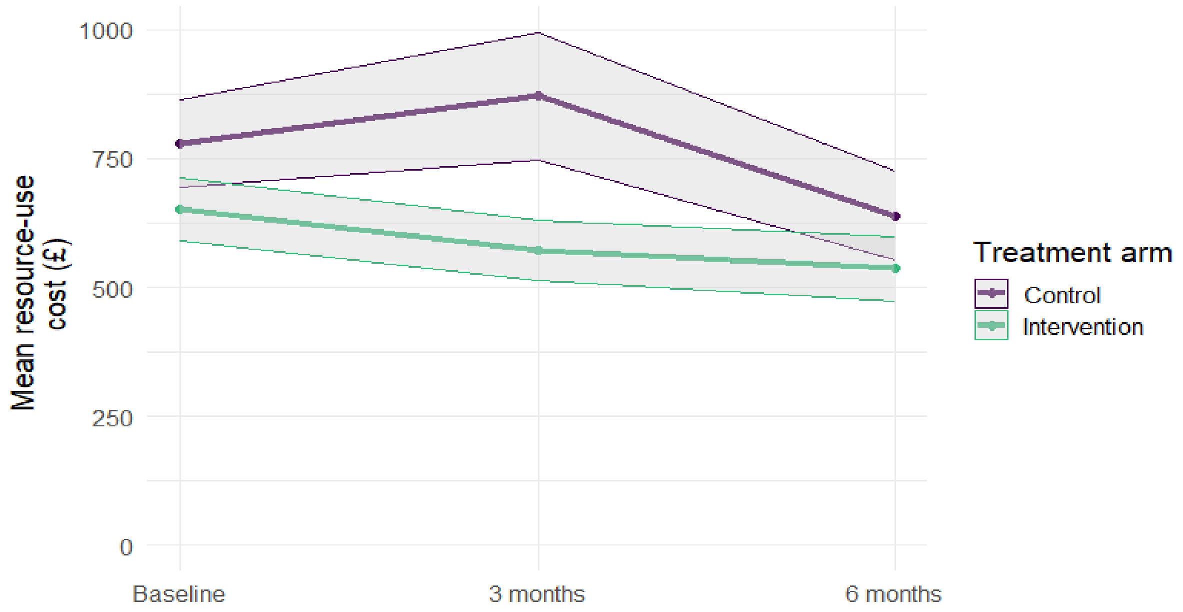

Figure S1. Mean resource use cost per participant over the trial period, by trial arm, with standard error shading (n = 117 control – with the outlier removed, n = 116 intervention). Source data are provided with this paper.

Table S3 presents this same data in numerical form, with the mean healthcare resource use at each time point for the intervention and control group (both unadjusted and adjusted for primary outcome selected, sex, age, index of multiple deprivation, ethnicity and all resource use at baseline). The mean difference between control and intervention group is included to illustrate the impact of removing the outlier on mean resource use at each time point, as the outlier was in the control arm. The data in the table indicates that the difference in resource use between groups at baseline is largely accounted for by this one outlier. However, at three months, the difference between groups is similar in the base case analysis and the scenario analysis with the outlier removed. This gives further confidence that controlling for resource use at baseline ensures that these baseline differences do not impact the cost-utility analysis results.

Table S3. Mean resource use over the trial period, by trial arm for the base case analysis and for the scenario analysis with the outlier removed.

Analysis	Visit	Control		Intervention		Difference	
		Unadjusted	Adjusted*	Unadjusted	Adjusted*	Unadjusted	Adjusted*
Base case	Baseline	893	1,203	652	965	241	238
	Three months	875	1,117	571	900	303	217
	Six months	656	424	536	377	119	47
Outlier removed	Baseline	779	1,130	652	1,001	127	128
	Three months	871	646	571	448	300	198
	Six months	639	215	536	178	103	37

*Adjusted for: primary outcome selected, sex, age, index of multiple deprivation, ethnicity and all resource use at baseline

Table S4 below shows the cost-effectiveness results with the outlier observation removed, and Figure S2 below presents the ICER planes and CEAC curves for both the NHS/PSS and societal perspectives, with the outlier observation removed from the analysis.

Similar to the base case analysis, the point estimate ICER for the NHS/PSS perspective is below both the £20,000/QALY and £30,000/QALY thresholds. Figure S2(b) shows that, at a threshold of £20,000/QALY, there would be approximately 68% probability of the intervention being considered cost-effective, increasing to 86% at a threshold of £30,000/QALY. While these probabilities are lower than in the base case analysis (with the outlier included), it is not likely that the interpretation of the cost-effectiveness analysis results would change based on inclusion or exclusion of this participant.

From a broader societal perspective, the intervention remains dominant with the exclusion of the outlier from the analysis. Figure S2(d) shows that there would be a 93% probability of the intervention being considered cost-effective at a threshold of £20,000/QALY, increasing to 96% at a threshold of £30,000/QALY, with the outlier excluded from the analysis. Further, Figure S2(c) and (d) indicate that the intervention may be cost-saving when a societal perspective is adopted, with a 73% probability of cost-saving at a threshold of £0/QALY. These results are very similar to the base case analysis, and thus, interpretation of the cost-effectiveness results is again unlikely to change based on inclusion or exclusion of this participant. There is no further indication to exclude this individual from the analysis, and thus the base case analysis did not exclude this participant.

Table S4. Incremental cost-effectiveness results with resource use outlier removed from the analysis

	Perspective	Pooled estimate	95% CI Lower limit	95% CI Upper limit
QALY difference		0.0485471	0.0109873	0.0861069
Cost difference (£)	NHS/PSS	767	439	1,095
	Societal	-536	-2,221	1,148
ICER	NHS/PSS	£15,803/QALY	£6,337/QALY	£65,514/QALY
	Societal	Dominant	Dominant	£43,548/QALY
INMB	NHS/PSS	689	-766	2,144
	Societal	1,894	-941	4,728

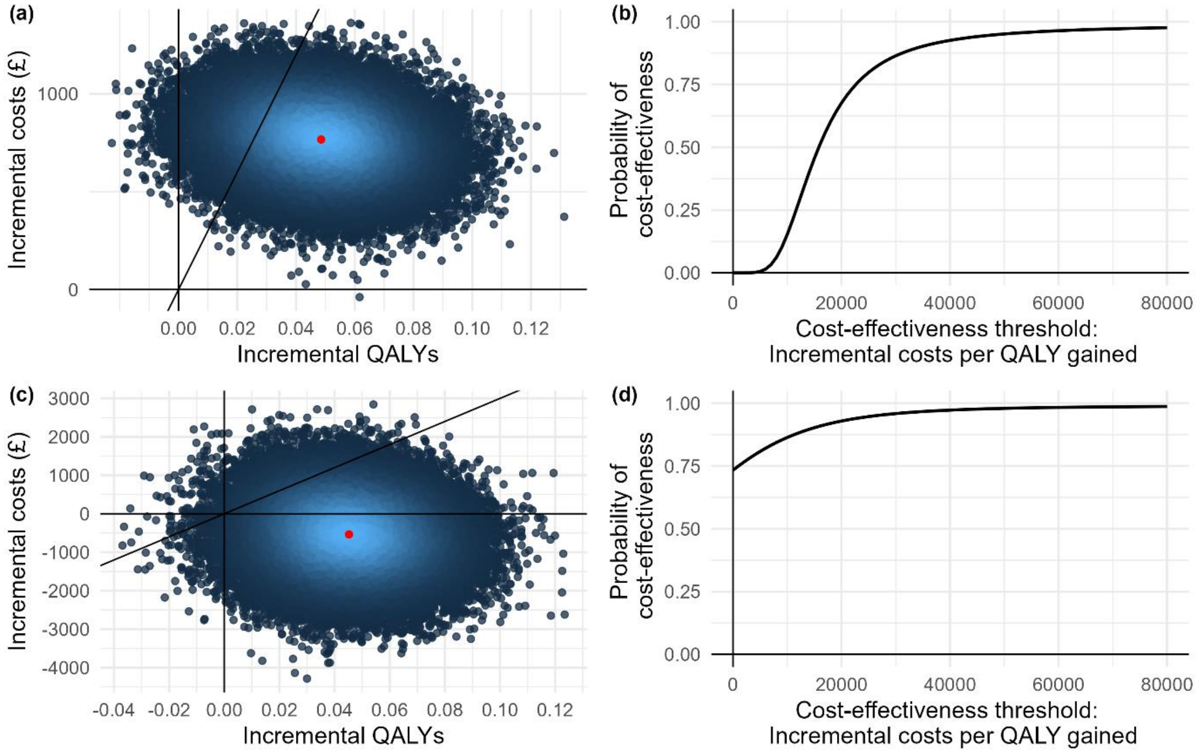

Figure S2. Incremental cost-effectiveness ratio (ICER) Planes and cost-effectiveness acceptability curves (CEACs) for the National Health Service and Personal Social Services (NHS/PSS) and Societal perspectives using 5,000 non-parametric bootstrap replicates on 10 imputed datasets from an original sample of n = 233 individuals (n = 117 control – with the outlier removed, n = 116 intervention). Each individual had three scheduled observations, with a total of 25/699 missing observations (1/351 missing observation in the control arm, 24/348 missing observations in the intervention arm). **(a)** ICER Plane from the NHS/PSS perspective (outlier removed). **(b)** CEAC from the NHS/PSS perspective (outlier removed). **(c)** ICER Plane from the societal perspective (outlier removed). **(d)** CEAC Plane from the societal perspective (outlier removed).
QALY: Quality-adjusted life year.

References

1. Husereau D, Drummond M, Augustovski F, de Bekker-Grob E, Briggs AH, Carswell C*, et al.* Consolidated Health Economic Evaluation Reporting Standards (CHEERS) 2022 Explanation and Elaboration: A Report of the ISPOR CHEERS II Good Practices Task Force. *Value in Health* **25**, 10-31 (2022).

2. Counterweight. Pricing. 2021. Accessed: 24 October 2023. Available from https://www.counterweight.org/pages/pricing

3. Jones KC, Weatherly H, Birch S, Castelli A, Chalkley M, Dargan A*, et al.* Unit Costs of Health and Social Care 2022 Manual.). Personal Social Services Research Unit (University of Kent) & Centre for Health Economics (University of York) (2023).

4. Little M, Gray A, Altman D, Benedetto U, Flather M, Gerry S*, et al.* Five-year costs from a randomised comparison of bilateral and single internal thoracic artery grafts. *Heart* **105**, 1237-1243 (2019).
