# Supplementary material for: Cost-effectiveness of the ReDIRECT/counterweight-plus weight management programme to alleviate symptoms of long COVID
Source: Nat Commun. 2025 Jul 1;16:5592. doi: 10.1038/s41467-025-59909-6 (PMC12218384; doi:10.1038/s41467-025-59909-6)
Supplement: Supplementary file 2 — Reporting Summary [file 41467_2025_59909_MOESM2_ESM.pdf]

nature portfolio

Corresponding author(s): Heather Lynne Fraser

Last updated by author(s): Apr 25, 2025

Reporting Summary

Nature Portfolio wishes to improve the reproducibility of the work that we publish. This form provides structure for consistency and transparency in reporting. For further information on Nature Portfolio policies, see our Editorial Policies and the Editorial Policy Checklist.

Statistics

For all statistical analyses, confirm that the following items are present in the figure legend, table legend, main text, or Methods section.

n/a	Confirmed	
☐	☒	The exact sample size (*n*) for each experimental group/condition, given as a discrete number and unit of measurement
☐	☒	A statement on whether measurements were taken from distinct samples or whether the same sample was measured repeatedly
☐	☒	The statistical test(s) used AND whether they are one- or two-sided<br>*Only common tests should be described solely by name; describe more complex techniques in the Methods section.*
☐	☒	A description of all covariates tested
☒	☐	A description of any assumptions or corrections, such as tests of normality and adjustment for multiple comparisons
☐	☒	A full description of the statistical parameters including central tendency (e.g. means) or other basic estimates (e.g. regression coefficient) AND variation (e.g. standard deviation) or associated estimates of uncertainty (e.g. confidence intervals)
☐	☒	For null hypothesis testing, the test statistic (e.g. *F*, *t*, *r*) with confidence intervals, effect sizes, degrees of freedom and *P* value noted<br>*Give P values as exact values whenever suitable.*
☒	☐	For Bayesian analysis, information on the choice of priors and Markov chain Monte Carlo settings
☒	☐	For hierarchical and complex designs, identification of the appropriate level for tests and full reporting of outcomes
☒	☐	Estimates of effect sizes (e.g. Cohen's *d*, Pearson's *r*), indicating how they were calculated

Our web collection on statistics for biologists contains articles on many of the points above.

Software and code

Policy information about availability of computer code

Data collection	Trial participants entered data directly into bespoke electronic Case Report Forms (eCRF). Screenshots of the ReDIRECT study eCRF questionnaires are available via Figshare at https://doi.org/10.6084/m9.figshare.21270837. Data relating to dietetic visits were entered in Excel spreadsheets.
Data analysis	All analyses were conducted using R Statistical Software, version 4.4.1 (2024-06-14 ucrt) and RStudio version 2024.04.2-764. The R packages used in the analysis include: boot (R package version 1.3-30), data.table (R package version 1.16.0), lme4 (R package version 1.1-35.5), mice (R package version 3.16.0), summarytools (R package version 1.0.1), ggpattern (R package version 1.1.4), readstata13 (R package version 0.10.1), viridis (package version 0.6.5), flextable (R package version 0.9.6), finalfit (R package version 1.0.8), systemfit (R package version 1.1-30), doBy (R package version 4.6.22), ggpubr (R package version 0.6.0), ggpointdensity (R package version 0.1.0), eq5d (R package version 0.15.3), table1 (R package version 1.4.3), miceadds (R package version 3.17), gtsummary (R package version 2.0.2), reshape (R package version 0.8.9), tidyverse (R package version 2.0.0), devtools (R package version 2.4.5), scales (R package version 1.3.0), rmarkdown (R package version 2.28), kableExtra (R package version 1.4.0).

For manuscripts utilizing custom algorithms or software that are central to the research but not yet described in published literature, software must be made available to editors and reviewers. We strongly encourage code deposition in a community repository (e.g. GitHub). See the Nature Portfolio guidelines for submitting code & software for further information.

Data

Policy information about availability of data

All manuscripts must include a data availability statement. This statement should provide the following information, where applicable:

- Accession codes, unique identifiers, or web links for publicly available datasets
- A description of any restrictions on data availability
- For clinical datasets or third party data, please ensure that the statement adheres to our policy

As per our study protocol, access to the raw data is restricted to the primary research team while the research is being conducted and prior to publication of the primary research papers. The primary research papers include the primary trial outcome paper, the health economics paper, the process evaluation paper and the Patient and Public Involvement paper. Upon publication of these papers, fully anonymized and minimized data (and data dictionaries) will be placed in a research data repository with access given to bona fide researchers, on request to the corresponding author and subject to appropriate data-sharing agreements. Proposals will be assessed on a monthly basis, with a response within 2 months of submission. The Trial Statistical Analysis Plan is available via ISRCTN (https://doi.org/10.1186/ISRCTN12595520), and ReDIRECT study eCRF questionnaire screenshots are available via Figshare at https://doi.org/10.6084/m9.figshare.21270837

Research involving human participants, their data, or biological material

Policy information about studies with human participants or human data. See also policy information about sex, gender (identity/presentation), and sexual orientation and race, ethnicity and racism.

Reporting on sex and gender	Table 1 in the Results section of the manuscript (Population characteristics in the two groups in the randomised trial) reports the sex of participants in the Control group, Intervention group and overall. This refers to the biological sex of participants (self-reported). Seemingly unrelated regressions used to estimate the Incremental Cost-Effectiveness Ratio (ICER) controlled for sex as a potential confounder, but results were not reported separately by sex. Regressions used to estimate adjusted cost and utility summary results controlled for sex, but results were not reported separately by sex.
Reporting on race, ethnicity, or other socially relevant groupings	Population characteristics in terms of ethnicity, index of multiple deprivation (IMD) and region were reported in the related trial manuscript reporting the primary trial outcomes (submitted and declared as a related manuscript in the initial article submission), titled Remotely-delivered weight management for people living with Long COVID and overweight: the randomised wait-list controlled ReDIRECT trial. This was cited in the submitted manuscript for reference. Regressions used to estimate adjusted cost and utility summary results controlled for ethnicity and IMD, but results were not reported separately by ethnicity or IMD. Seemingly unrelated regressions used to estimate the Incremental Cost-Effectiveness Ratio (ICER) controlled for IMD as a potential confounder, but results were not reported separately by IMD.
Population characteristics	Table 1 in the Results section of the manuscript (Population characteristics in the two groups in the randomised trial) reports the age, sex, mean weight, mean body mass index, utility scores and employment status at baseline. Further detail on population characteristics, including systolic and diastolic blood pressure, hypertension, Type 2 diabetes mellitus, mean time since first COVID infection, vaccination status and number of medications taken, were reported in the related trial manuscript reporting the primary trial outcomes (submitted and declared as a related manuscript in the initial article submission), titled Remotely-delivered weight management for people living with Long COVID and overweight: the randomised wait-list controlled ReDIRECT trial. This was cited in the submitted manuscript for reference.
Recruitment	We aimed to recruit a total of 240 people aged 18 years and above with self-reported symptoms of Long COVID and overweight/obesity from general practice, social media, newspaper advertisements and existing networks, such as national Long COVID groups. Full details on recruitment, including efforts to ensure a representative study population, are reported in the related trial manuscript reporting the primary trial outcomes (submitted and declared as a related manuscript in the initial article submission), titled Remotely-delivered weight management for people living with Long COVID and overweight: the randomised wait-list controlled ReDIRECT trial, and in the trial protocol paper. These were cited in the submitted manuscript for reference.
Ethics oversight	Ethical approval was obtained from the South-East Scotland Research Ethics Committee 01 (REC reference number: 21/SS/0077).

Note that full information on the approval of the study protocol must also be provided in the manuscript.

Field-specific reporting

Please select the one below that is the best fit for your research. If you are not sure, read the appropriate sections before making your selection.

☐ Life sciences ☒ Behavioural & social sciences ☐ Ecological, evolutionary & environmental sciences

For a reference copy of the document with all sections, see nature.com/documents/nr-reporting-summary-flat.pdf

Behavioural & social sciences study design

All studies must disclose on these points even when the disclosure is negative.

Study description	Economic evaluation (cost-utility analysis) alongside a randomised controlled trial, using quantitative data.
Research sample	Participants, male and female, were recruited across the UK via social media, online forums, Long COVID networks, newspaper adverts, and primary care records. Eligible participants, aged ≥18 years, had a self-reported body mass index (BMI) >27kg/m2 (>25kg m^2 for South Asians), self-reported LC symptoms for >12 weeks before first recruitment contact, and were not hospitalised, currently or for over 10 days during acute COVID-19 infection.<br>A total of 234 participants were recruited and enrolled with n=118 in the control arm and n=116 in the intervention arm. Mean age was 46.1 in the control arm and 46.4 in the intervention arm. In the control arm, 85.6% of participants were female, with 85.3% of participants in the control arm female. Mean weight in both arms was 102kg. To the best of our knowledge, the sex distribution in our study sample is representative of the sex distribution of the long COVID population. Efforts were made to recruit participants representative of ethnicity distribution in the population of England, Scotland and Wales.
Sampling strategy	Assuming the SD of the primary outcome at follow-up (derived from the symptom score at follow-up, standardised using the same mean and SD as used at baseline) is one, then to have 90% power to detect a between group difference of 0.5 at follow-up, at a 5% significance level, requires a sample size of 86 per group with follow-up data. To allow for attrition, we aimed to randomise 240 people.<br>We aimed to recruit a total of 240 people aged 18 years and above, with self-reported symptoms of Long COVID and overweight/obesity (n = 120 per study arm) from general practice, social media, newspaper advertisements and existing networks, such as national Long COVID groups. A positive COVID-19 diagnosis was not a requirement for this study due to the unavailability of COVID-19 tests at the start of the pandemic. This inclusive approach was chosen based on feedback from the PPI group and a strong steer from the funder.
Data collection	Trial outcomes were self-measured remotely to minimise participant burden and maximise retention. Web-based questionnaires collected self-reported baseline characteristics and sociodemographic data (including sex), self-measurements (using digital scales (A&D Medical Model UC-502) and automated blood pressure monitors (Kinetik Wellbeing Model - WBP1)), Long COVID symptoms, healthcare resource use, productivity (employment), out-of-pocket costs, alternative treatments, health-related quality of life (HRQoL), and psychological outcomes. Participants entered data directly into bespoke electronic Case Report Forms (eCRF), verified by research staff, or entered by research staff following communication via email, text, or phone. Additional data were captured in Excel spreadsheets by dietitians (screening/programme start dates, withdrawal date and reason, programme switched to and date, number of sachets) and securely transferred to the biostatisticians.<br>Due to the nature of the diet intervention, participants, researchers, and the dietitians who delivered the intervention were aware of group allocations. Statisticians analysing the data remained blinded until the statistical analysis plan was completed and the primary analysis database locked.
Timing	December 2021 to March 2023.
Data exclusions	No data were excluded from the analysis.
Non-participation	Applicants for inclusion (n=297) were screened for enrolment, and n=240 recruited and consented between 23 December 2021 and 04 July 2022; five did not complete the baseline assessment and were not randomised, and one withdrew consent after randomisation, resulting in n=234 randomised to the treatment (n=116) and wait-listed control (n=118) groups.<br>In the intervention arm, two (0.8%) participants withdrew at three months, and one (0.4%) withdrew at six months. The reasons for withdrawals were pregnancy, unknown reasons and emigration. In addition, 9 (4%) participants were lost to follow-up at three months and 7 (3%) at six months. All but one participant lost to follow-up (at six months) were in the intervention arm. Data were available for the primary outcome at three months for 219 participants (94%, n=101 in the intervention group, n=118 in the delayed-entry control group) and at six months for 214 participants (91%, n=97 intervention, and n=117 control).
Randomization	Participants were allocated to intervention or control groups using a mixed minimisation and randomisation approach to balance the groups with respect to the participant-selected dominant Long COVID symptom (fatigue, breathlessness, pain, anxiety/depression, or other), sex (male, female, other), age (<50, 50+), ethnicity (White, South Asian, other), and index of multiple deprivation (postcode-based, deciles 1-5, 6-10). Randomisation took place after the completion of baseline assessments, using an online system developed and maintained by the Robertson Centre for Biostatistics (University of Glasgow, UK).

Reporting for specific materials, systems and methods

We require information from authors about some types of materials, experimental systems and methods used in many studies. Here, indicate whether each material, system or method listed is relevant to your study. If you are not sure if a list item applies to your research, read the appropriate section before selecting a response.

Materials & experimental systems

n/a	Involved in the study	
☒	☐	Antibodies
☒	☐	Eukaryotic cell lines
☒	☐	Palaeontology and archaeology
☒	☐	Animals and other organisms
☐	☒	Clinical data
☒	☐	Dual use research of concern
☒	☐	Plants

Methods

n/a	Involved in the study	
☒	☐	ChIP-seq
☒	☐	Flow cytometry
☒	☐	MRI-based neuroimaging

Clinical data

Policy information about clinical studies

All manuscripts should comply with the ICMJE guidelines for publication of clinical research and a completed CONSORT checklist must be included with all submissions.

Clinical trial registration

ISRCTN registry 12595520.

Study protocol

Haag L. et al,. NIHR Open Research 2023; 2:57. https://doi.org/10.3310/nihropenres.13315.2
Ethical approval of the study protocol was obtained from the South-East Scotland Research Ethics Committee 01 (reference number: 21/SS/0077).

Data collection

Participants were recruited and consented between 23 December 2021 and 04 July 2022. Data was collected remotely, from participants across the UK, using electronic case report forms.

Outcomes

The primary outcome was a continuous measure derived from the symptom score for the most important Long COVID symptom, selected by each participant at baseline (fatigue, breathlessness, pain, anxiety/depression, or other). Symptom scores were assessed using validated questionnaires for the core symptoms of fatigue (Chalder Fatigue Scale), breathlessness (MRC Dyspnoea Scale), pain (P4 Pain Rating Scale), anxiety and depression (Hospital Anxiety and Depression Scale). Other symptoms could be added via free text boxes and were scored using a 10-point visual analogue scale (VAS).

Secondary outcomes included the core Long COVID symptoms (fatigue, breathlessness, pain, anxiety/depression), all 'other' Long COVID symptoms reported, self-measured weight, height, blood pressure, health-related quality of life, Work Productivity and Activity Impairment, healthcare resource use, medication (prescribed and over-the-counter) and food/drink costs for the previous week.

Plants

Seed stocks

Not applicable.

Novel plant genotypes

Not applicable.

Authentication

Not applicable.
